# Engineering lentiviral vectors for modulation of dendritic cell apoptotic pathways

**DOI:** 10.1186/1743-422X-10-240

**Published:** 2013-07-20

**Authors:** James CM Wang, Tânia C Felizardo, Bryan CY Au, Daniel H Fowler, Gregory A Dekaban, Jeffrey A Medin

**Affiliations:** 1 University Health Network, Canadian Blood Services Building, 67 College St. Room 406, Toronto, Ontario M5G2M1, Canada; 2 Faculty of Medicine, University of Toronto, Toronto, ON M5S 1A8, Canada; 3 Experimental Transplantation and Immunology Branch, National Cancer Institute, National Institute of Health, Bethesda, MD 20892, USA; 4 Department of Microbiology and Immunology, Western University, London, ON N6A 5C1, Canada; 5 BioTherapeutics Research Laboratory, Robarts Research Institute, London, ON N6A 5K8, Canada; 6 Institute of Medical Science and the Department of Medical Biophysics, University of Toronto, Toronto, ON M5S 1A8, Canada

**Keywords:** HER-2/neu, Lentivirus, Dendritic cell vaccine, Dendritic cell longevity/lifespan

## Abstract

**Background:**

Dendritic cells (DCs) are promising mediators of anti-tumor immune responses due to their potent antigen-presentation capacity. Unfortunately, cancer cells can often disarm differentiated DCs by rendering them incapable of maturation or by promoting their apoptosis. DC vaccine regimens attempt to generate functional DCs and preload them with Tumor-Associated Antigens (TAAs) to target various malignancies. Despite these efforts, the efficacy of DC vaccines in clinical trials is still rather disappointing to date. In addition to undergoing cancer-induced apoptosis, it is well established that DCs are intrinsically short-lived cell types. It is likely that a significant portion of infused DCs undergo apoptosis prior to locating and activating naïve TAA-reactive T cells.

**Methods:**

In our current study, we constructed and investigated novel bicistronic lentivectors (LVs) encoding the cDNA for the xeno-TAA*,* rat HER-2/neu (rHER-2), along with five candidate mouse DC survival factors (c-FLIP_S_, c-FLIP_L_, Bcl-_XL_, M11L, and AKT-1) that operate in both the extrinsic and intrinsic cycles of apoptosis. The murine DC cell line, DC2.4 was transduced separately with each novel LV construct. Infected cells were enriched via flow cytometric methods based on rHER-2 expression. Transduced DC2.4 cell lines were then exposed to Fetal Calf Serum (FCS) withdrawal and to specific pharmacological apoptosis-inducing agents. DC2.4 cell death was assayed based on Annexin V and PI double-positive staining via flow cytometry. The phenotype and function of transduced DC2.4 cells and primary bone marrow-derived DCs were then assessed via expression and secretion of DC markers and cytokines, respectively.

**Results:**

DC2.4 cells transduced with LVs encoding cDNAs for c-FLIP_S_, c-FLIP_L_, Bcl-_XL_, and M11L were protected from apoptosis when exposed to low FCS-containing culture media. When treated with an anti-CD95 antibody, only DC2.4 cells transduced with LVs encoding c-FLIP_S_ and c-FLIP_L_ were protected from apoptosis. In contrast, only DC2.4 cells transduced with LVs encoding Bcl-_XL_ and M11L were protected from effects of staurosporine (STS) treatment. Also, LV-modified DCs maintained their original phenotype and function.

**Conclusions:**

We present evidence that by employing novel recombinant bicistronic LVs we can simultaneously load DCs with a relevant TAA and block apoptosis; thereby confirming the usage of such LVs in the modulation of DC lifespan and function.

## Background

Dendritic cells (DCs) function to initiate and orchestrate the adaptive immune response to pathogens as well as to suppress self immune responses in their absence. To perform these roles, DCs are regulated by both external and internal signaling factors. External factors include IL-10, which has been shown to lower DC survival by suppressing anti-apoptotic genes *Bcl-2*, *Bcl-*_*XL*_, and *Bfl-1*[1]*.* In addition to being influenced by external factors promoting cell death, DCs are intrinsically short-lived cell types [2]. Kinetic studies have shown that antigen-bearing mature DCs undergo apoptosis after only 2–3 days *in vitro* and *in vivo*, even in the absence of an immunosuppressive niche [3,4]. In the context of infections, the short lifespan of DCs is still sufficient to permit eradication of most pathogens owing to the plethora of pathogen-specific T cell clones in the periphery; in fact, DC’s short lifespan probably evolved to prevent autoimmunity. However, in the context of immunotherapy, short-lived, *ex vivo*-modified DC vaccines likely undergo apoptosis prior to encountering and optimally-activating TAA-reactive T cell clones. Hence, the short lifespan of DCs may be a factor in the relatively disappointing results of many DC vaccine-based clinical trials to date and may be a major hindrance to mounting potent immune responses that result in objective cancer regressions [5].

Two major pathways are responsible for the initiation of apoptosis in general: the extrinsic and intrinsic pathways [6]. The extrinsic pathway is triggered when cell surface receptors are activated by pro-apoptotic ligands [7]. Cell surface death receptors including Death Receptor 4 (DR4), DR5, and CD95 (Fas-receptor) are activated by their ligands TRAIL and Fas-ligand, respectively. Once death receptors are activated, their death domains bind to an adaptor protein known as Fas-Associated Death Domain (FADD), which recruits procaspase-8 dimers to form a death-inducing signaling complex (DISC) [8]. Upon formation of DISC, procaspase-8 then undergoes autocatalytic cleavage into caspase-8 (FLICE). Caspase-8 may then initiate the cleavage and activation of procaspases −3, −6, and −7, whereupon the intrinsic and extrinsic cycles of apoptosis converge. A negative regulator of the extrinsic cycle of apoptosis is the caspase-8 homologue, Cellular FLICE Inhibitory Protein (c-FLIP) [9,10]. As its name suggests, c-FLIP functionally inhibits caspase-8 by competitively binding to FADD and preventing recruitment of more caspase-8 molecules. The two main isoforms of c-FLIP, the long and short forms, are capable of intervening at different checkpoints of procaspase-8 processing and both may play a role in extending DC longevity [9,11].

The intrinsic cycle of apoptosis is activated in response to cellular signals triggered during DNA damage and by other forms of cell stress [7]. Cell stress, in turn, activates the tumor suppressor protein p53, leading to further activation of upstream pro-apoptotic molecules Puma and Noxa along with downstream pro-apoptotic molecules Bax and Bak. The intrinsic cycle is marked by the eventual disruption of the mitochondrial membrane potential and subsequent release of cytochrome c from mitochondria following which apoptosis ensues. Bcl-2 and Bcl-_XL_ function to antagonize the action of Bax and Bak and hence may be considered as anti-apoptotic molecules themselves and thus appear to be important players in DCs [11-13].

In addition to endogenous anti-apoptotic molecules, viral homologues affecting similar cellular functions have also been described. In particular, M11L is a myxoma virus-derived structural and functional homolog of Bcl-2. We have shown previously that M11L possesses potent anti-apoptotic properties by acting in the same pathway as Bcl-2 and Bcl-_XL_[14]. Unlike their cellular counterparts Bcl-2 and Bcl-_XL_, which have been shown to be converted to proapoptotic molecules upon caspase cleavage, viral homologs appear to be resistant to caspase cleavage and such conversions [15]. Owing to this property, they may demonstrate more robust anti-apoptotic effects without activating negative feedback mechanisms that result in their conversion into apoptotic factors.

Lentivectors (LVs) are promising vehicles for gene delivery due to their ability to integrate into the host cell genome and to transduce both dividing and non-dividing cells [16]. In the context of DC vaccines, LVs permit high TAA gene transfer efficiencies and expression [17], the generation of a broad repertoire of presented peptides, the induction of more stable peptide-MHC complexes, and the ability to co-express multiple genes such as TAAs and survival factors [18]. Along these lines we previously demonstrated that LVs encoding the sequence for the xeno-TAA (rat) HER-2 efficiently transduced murine bone marrow-derived DCs [19]. Furthermore, when these transduced DCs were injected into mice harboring murine HER-2-positive tumors, potent systemic CD4 and CD8 T cell responses along with humoral responses were generated that significantly inhibited tumor growth [19].

In our current study, we demonstrate that LV-engineered expression of a number of survival factors that work on both the extrinsic and intrinsic pathways to modulate DC apoptosis. This work has implications for the development of durable and efficacious immunotherapy based on sustained TAA presentation using these very potent antigen-presenting cells.

## Results

### Construction and validation of novel bicistronic LVs encoding rHER-2 and survival factors

**Figure 1:**
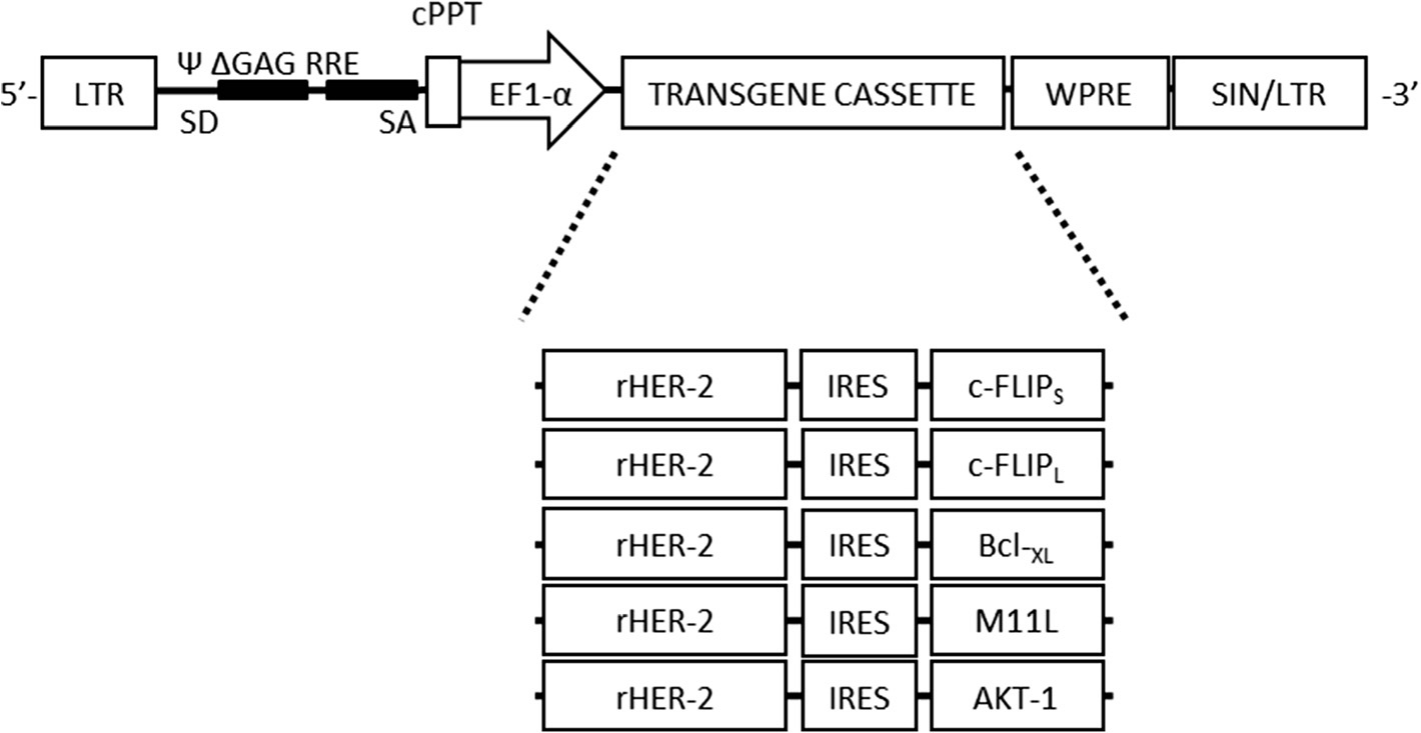
**Schematic of novel bicistronic LVs encoding rHER-2 and survival factors.** Illustration of self-inactivating (SIN) lentiviral transfer vectors. Map highlights essential vector elements; survival factors include: c-FLIP_S_, c-FLIP_L_, Bcl-_XL_, M11L, and AKT-1. Expression of proteins of interest is driven by the constitutive promoter, EF-1α. The IRES from the EMCV enables the expression of the secondary cDNA encoding the survival factors.

We constructed recombinant bicistronic lentivectors incorporating rHER-2 and second transgenes encoding one of five candidate anti-apoptotic, survival factors: c-FLIP_S_, c-FLIP_L_, Bcl-_XL_, M11L, and AKT-1 to target either the extrinsic or intrinsic cycle of apoptosis (Figure 1). These LVs are denoted LV/rHER-2.c-FLIP_S_, LV/rHER-2.c-FLIP_L_, LV/rHER-2.Bcl-_XL_, LV/rHER-2.M11L, and LV/rHER-2.AKT-1, respectively. We selected the potent oncogene *AKT-1* as a positive control for our anti-apoptosis experiments as it has been shown that, while isoforms AKT-1 and AKT-2 are present in hematopoietic cells, AKT-1 is the predominant isoform found in DCs [20]. The encephalomyocarditis Virus (EMCV) Internal Ribosomal Entry Site (IRES) element was subcloned into the LV backbone to facilitate the expression of the downstream survival factor transgene product.

**Figure 2:**
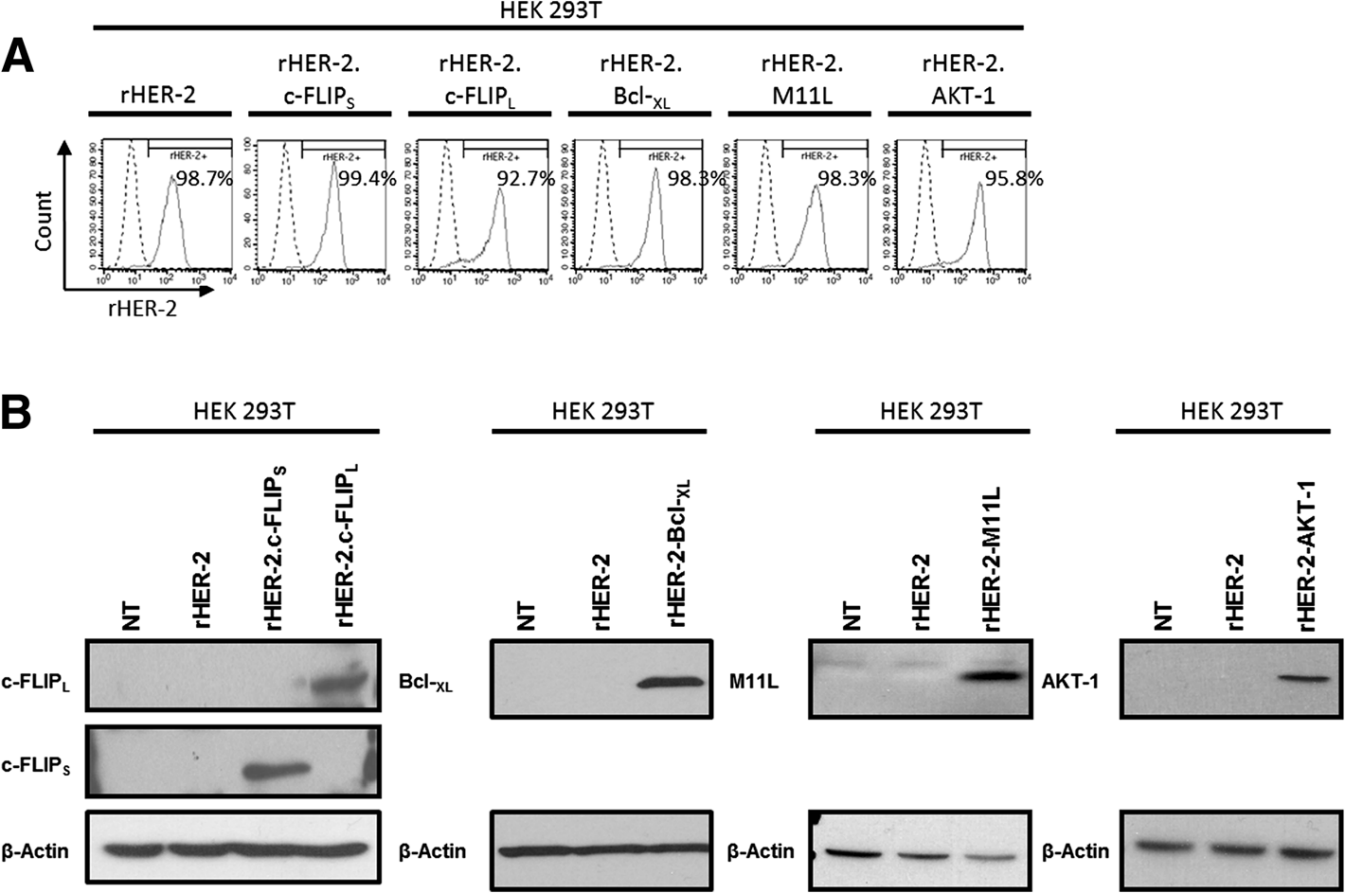
**Validation of novel bicistronic LVs encoding rHER-2 and survival factors. A)** Flow cytometry plots illustrating expression of rHER-2 in transduced HEK 293T cells. **B)** Enforced over-expression of survival transgenes is confirmed by immuno-blotting of protein extracts from transduced HEK 293T cells.

Concentrated LV stocks were produced as before [19]. To validate our novel LVs, we first transduced HEK 293T cells at a multiplicity of infection (MOI) of 20 (with titer estimated from earlier test transductions; data not shown) and assessed rHER-2 transgene expression by flow cytometry (Figure 2A). As expected, HEK 293T cells were transduced at high efficiencies (ranging from 92.7% to 99.4% rHER-2-positive) and expressed high amounts of rHER-2 TAA even with these complex constructs. Next, transduced populations were collected, lysates generated, and extracts analyzed by immuno-blotting for increased expression of survival factors (Figure 2B). Transduced HEK 293T cell lines expressed large quantities of the viral Bcl-2 homolog, M11L, along with wild-type c-FLIP_S_, c-FLIP_L_, Bcl-_XL_, and AKT-1 above endogenous basal levels.

### Transduction of the DC2.4 murine DC cell line resulted in stable genetic modifications

**Figure 3:**
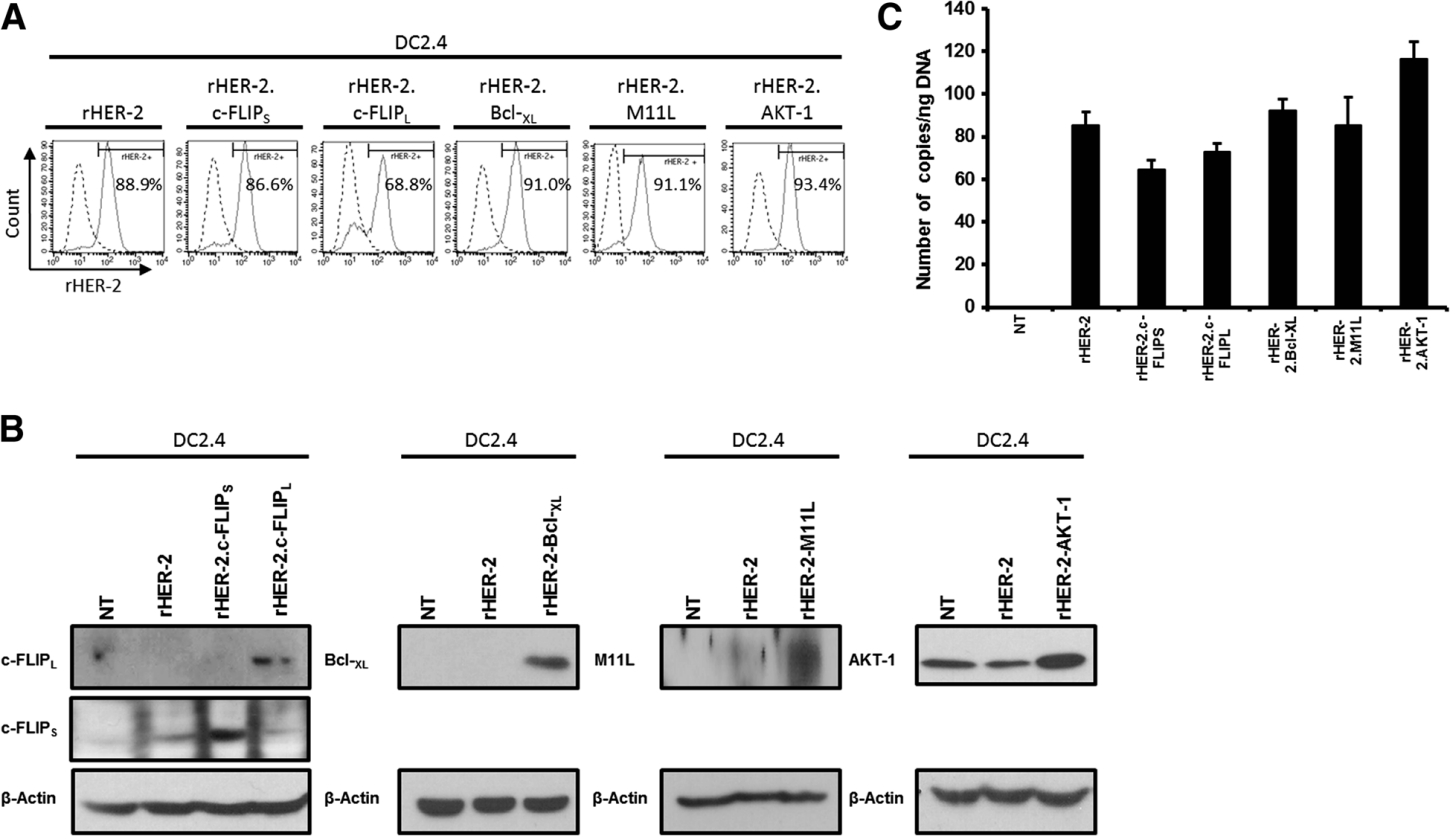
**Stable genetic modification of the DC2.4 cell line. A)** Flow cytometry plots illustrating expression of rHER-2 in LV-transduced DC2.4 cells. **B)** Enforced expression of survival genes is shown by immuno-blotting of protein extracts from transduced DC2.4 cells. **C)** Transduced DC2.4 cells were analyzed for WPRE copy numbers per ng of genomic DNA to assess proviral integration. Each DNA sample was tested in triplicate and mean values are shown. Experiment was performed once with error bars representing standard deviations.

Next, we sought to examine outcomes following transductions of the murine bone marrow-derived DC cell line, DC2.4 [21]. DC2.4 cells were transduced at an estimated MOI of 20 and sorted via flow cytometry based on surface rHER-2 expression. Post-sort population pools of transduced DC2.4s ranged from 68.8% to 93.4% rHER-2-positive (Figure 3A). As above, we performed immuno-blotting to ensure that transduced DC2.4 cells were over-expressing the various survival factors (Figure 3B). Generated transduced DC2.4 cell lines are hereafter referred to as: DC2.4/rHER-2.c-FLIP_S_, DC2.4/rHER-2.c-FLIP_L_, DC2.4/rHER-2.Bcl-_XL_, DC2.4/rHER-2.M11L, and DC2.4/rHER-2.AKT-1, respectively. We then performed Real-Time PCR to confirm stable LV integration and to demonstrate that transduced cells had similar proviral copies per construct. To this end, we utilized a method we previously developed [22] to probe for the exogenous Woodchuck hepatitis Post-transcriptional Regulatory Element (WPRE) (Figure 1), which is integrated into the host genome along with the transgenes of interest at a 1:1 ratio. As shown, average vector copy numbers for the DC2.4 cell lines ranged from ~60 to 110 copies/ng of genomic DNA (Figure 3C).

### Transduced DC2.4 cell lines are protected from FCS withdrawal-mediated non-specific induction of apoptosis pathways

**Figure 4:**
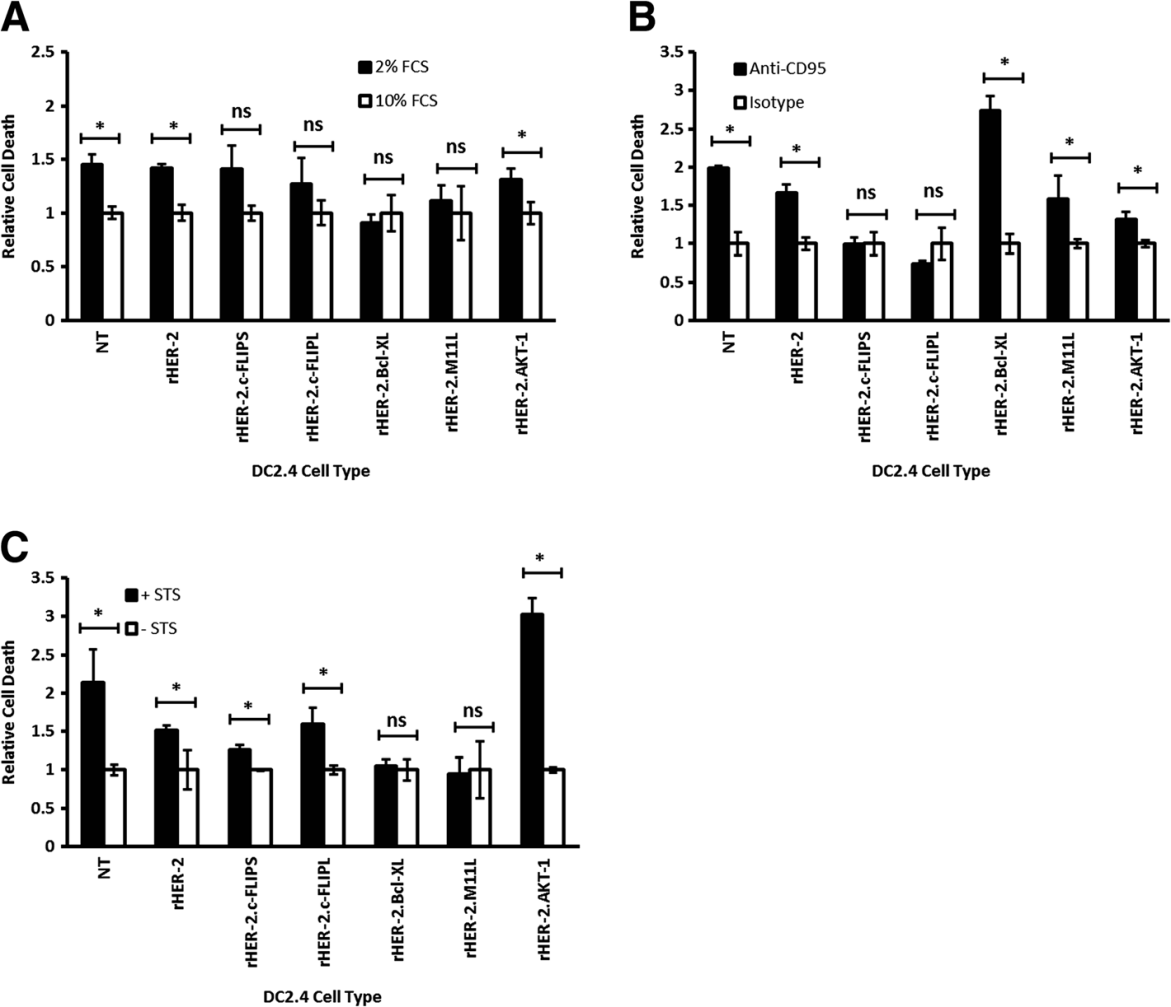
**Enforced expression of survival factors protect DC2.4 cells from targeted apoptosis. A)** DC2.4 cell lines were cultured in either 2% FCS or 10% FCS-containing media to assess the protective effect of survival factors. **B)** DC2.4 cell lines were treated with either anti-CD95 or isotype control antibodies to assess the protective effect of survival factor expression on activation of the extrinsic cycle of apoptosis. **C)** DC2.4 cell lines were treated with or without STS to assess the protective effect of survival factors from the activation of the intrinsic cycle of apoptosis. Cell death in all experiments was analyzed by Annexin V/PI flow cytometry assay and values were normalized to apoptotic cell death (Annexin V+/PI+ double-positive populations) observed in 10% FCS-containing media. Each treatment group was performed in triplicate. Each experiment was performed at least twice independently and representative data are shown with error bars representing standard deviations. *p values <0.05 were designated as significant (*).

To assay for the anti-apoptotic effects of survival factors, we first titrated and tested the inherent survival ability of DC2.4 cell lines by culturing cells in media containing decreased concentrations of FCS. We reasoned that induction of an intermediate starvation mode would highlight the protective effects of survival factors without exceeding their anti-apoptotic capacities. We then compared apoptotic cell death as measured by Annexin V+/PI+ double-positive staining and analyzed by flow cytometry DC2.4 cell lines cultured in decreasing concentrations of FCS (10% to 0%). Here, we found that in 2% FCS-containing media, the DC2.4 cell lines were under an intermediate starvation mode as defined by marked populations of cell death and cell survival (data not shown). Next, we proceeded to assess apoptosis in all the DC2.4 cell lines cultured in 10% and 2% FCS-containing media. Relative cell death was defined by Annexin V+/PI+ double-positive populations and apoptotic cell death for each cell line was normalized to the respective 10% FCS-containing treatment groups. As expected, non-transduced (NT) DC2.4 (DC2.4/NT) and LV/rHER-2- transduced DC2.4 (DC2.4/rHER-2) control cell lines that lack LV-engineered expression of survival factors exhibited significant reductions in viability between cells cultured in 2% versus 10% FCS-containing media (Figure 4A). On the contrary, DC2.4/rHER-2.c-FLIP_S_, DC2.4/rHER-2.c-FLIP_L_, DC2.4/rHER-2.Bcl-_XL_, and DC2.4/rHER-2.M11L lines all showed no significant differences in cell death between cells cultured in 2% versus 10% FCS-containing media. Unexpectedly, DC2.4/rHER-2.AKT-1 cells cultured in 2% FCS-containing media showed significantly increased cell death in comparison to the same cells cultured in 10% FCS-containing media, similar to cells expressing no survival factors. These results highlight the ability of c-FLIP_S_, c-FLIP_L_, Bcl-_XL_, and M11L to protect DC2.4s from cell death induced by FCS withdrawal.

### Transduced DC2.4 cell lines are protected from anti-CD95- and STS- mediated specific induction of apoptosis pathways

Next, we sought to employ a targeted approach to induce apoptosis in our engineered cell lines in order to evaluate the effects of enforced expression of the survival factors. Firstly, we treated our DC2.4 cell lines with either an anti-CD95 antibody or an isotype control antibody to engage the extrinsic cycle of apoptosis. As seen for the FCS withdrawal experiment above, DC2.4/NT and DC2.4/rHER-2 lines demonstrated increased cell death upon anti-CD95 treatment (Figure 4B). Likewise, DC2.4/rHER-2.Bcl-_XL_, DC2.4/rHER-2.M11L, and DC2.4/rHER-2.AKT-1 cell lines also exhibited increased cell death when treated with anti-CD95 antibody compared to the same cell lines treated with the isotype control. In alignment with known molecular pathways, DC2.4/rHER-2.c-FLIP_S_ and DC2.4/rHER-2.c-FLIP_L_ cells exhibited no difference in cell death when exposed to anti-CD95 antibody compared to the isotype control. Evidently both c-FLIP isoforms were capable of protecting transduced DC2.4s from the extrinsic cycle of apoptosis, while other survival factors, namely Bcl-_XL_, M11L, and AKT-1 were not.

Secondly, we treated transduced and control DC2.4 cell lines with STS, which selectively activates the intrinsic cycle of apoptosis [23]. As expected, only DC2.4/rHER-2.Bcl-_XL_ and DC2.4/rHER-2.M11L showed insignificant differences in cell death between the STS-treated and non-treated cells, while NT DC2.4s and DC2.4s transduced with other vectors failed to protect DC2.4 cells from STS-mediated induction of the intrinsic cycle of apoptosis (Figure 4C).

### LV transduction does not alter DC2.4 cell line and primary bone marrow-derived DC phenotypes or functions

**Figure 5:**
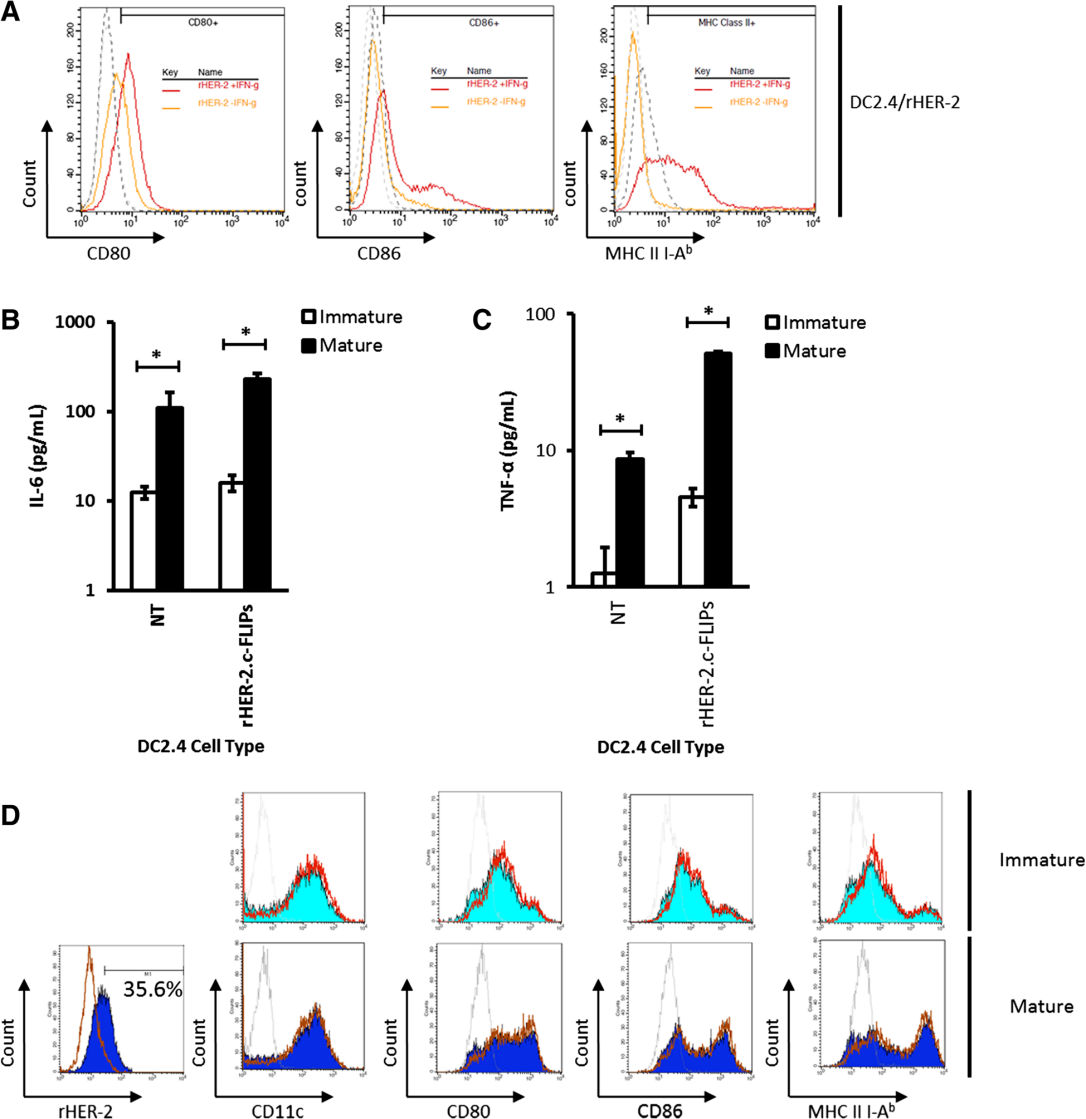
**LV transduction does not alter phenotypic and functional properties of DC2.4s and BMDCs. A)** DC2.4/rHER-2 cells were treated with recombinant mIFN-γ to induce maturation. Light and dark grey dotted lines represent isotype antibody controls in samples without and with IFN-γ treatment, respectively. Orange and red solid lines represent CD80, CD86, and MHC II-A^b^ stained samples without and with mIFN-γ treatment, respectively. **B**, **C)** Culture supernatant was collected 24 hours after maturation with mIFN-γ and analyzed for mIL-6 and mTNF-α secretion by ELISA. White bars represent immature DC2.4s (no mIFN-γ treatment) and black bars represent mature DC2.4s (mIFN-γ treatment). Cytokine levels were reported in pg/mL of supernatant. Each treatment group was performed in triplicate. Each experiment was performed twice independently and representative data are shown with error bars representing standard deviations. *p values <0.05 were designated as significant (*). **D)** BMDCs were transduced with LV/rHER-2.c-FLIP_S_ at MOI of 20 and analyzed for rHER-2, CD11c, CD80, CD86, and MHC II-A^b^ expression with and without recombinant mTNF-α-induced maturation. Solid red line represents NT cells and light and dark blue shaded histograms represent immature and mature transduced BMDCs, respectively. Light and dark grey lines represent isotype antibody control stainings of transduced immature and mature BMDCs, respectively. This experiment was representative of three independent experiments.

To further determine the clinical utility of our novel vectors, we next assessed the effects of LV transduction using the LV/rHER-2.c-FLIP_S_ vector as proof-of-principle. Firstly, we found that DC2.4 cells were effectively matured using recombinant murine IFN-γ *in vitro.* Here, we showed that DC2.4/rHER-2 cells treated with IFN-γ expressed higher levels of CD80, CD86, and MHC Class II compared to untreated control cells as determined by mean fluorescence intensity (MFI) on flow cytometry analyses, representative of a mature phenotype (Figure 5A). Next, we sought to determine if LV/rHER-2.c-FLIP_S_-transduced DC2.4 cells are functionally indistinct from NT cells. We found that transduced DC2.4 cells are morphologically indistinguishable from NT cells (data not shown) and, importantly, transduced DC2.4 cells were also functionally intact. Both DC2.4/NT and DC2.4/rHER-2.c-FLIP_S_ cells showed significant increases in IL-6 and TNF-α secretion upon maturation (Figure 5B, C). Lastly, we were interested in validating our vectors in primary bone marrow-derived DCs (BMDCs). Here, we transduced BMDCs with our LV/rHER-2.c-FLIP_S_ vector and achieved 35.6% transduction efficiency (Figure 5D). BMDCs were then matured using recombinant murine TNF-α and we found that upon maturation, BMDC/rHER-2.c-FLIP_S_ cells exhibited an immuno-phenotype profile indistinguishable from BMDC/NT cells, as determined by CD11c, CD80, CD86, and MHC Class II expression before and after maturation (Figure 5D). As expected, mature BMDC/NT and BMDC/rHER-2.c-FLIP_S_ cells exhibited increased levels of expression (based on MFI from flow cytometry analyses) of the maturation markers CD80, CD86, and MHC Class II compared to their immature counterparts.

## Discussion

Despite significant progress in the field of DC vaccine research, consistent anti-tumor immune responses that impact clinical outcomes remain elusive [5]. DCs possess innate properties that are normally crucial for maintaining immune system homeostasis but properties such as their relatively short lifespan can actually hinder their function in anti-cancer and anti-pathogen therapy.

In this report we aimed to engineer expression of a relevant TAA and to modulate DC apoptosis pathways using a bicistronic LV-mediated gene delivery approach. LVs encoding TAAs may effectively induce continuous DC production of TAAs and trigger endogenous processing and presentation of peptides on MHC molecules. As a result, the generated peptide-MHC complexes are more stable relative to those generated from conventional peptide-pulsing techniques [24].

We previously demonstrated the utility of loading DCs, employing this transduction mechanism using a LV encoding the cDNA for rHER-2, to generate immune responses targeted against tumors expressing murine HER-2 [19]. There we were successful in attaining objective anti-tumor responses that correlated with surrogate markers such as CD4+ and CD8+ T cell responses and humoral responses [19]. We observed significant numbers of CD8+/CD4+ T cells that secreted IFN-γ in the presence of mHER-2-positive tumors as well as CD4+ T cell infiltration at the tumor site. Furthermore, a significant humoral immune response was generated as indicated by elevated anti-HER-2 antibodies in the serum. Lastly, adoptive transfer of splenocytes into naïve mice illustrated immunological memory. Yet, in spite of such early tumor control and encouraging surrogate outcomes, RM-1-mHER-2 subcutaneously-implanted tumors eventually grew out in the treated mice [19]. Such outcomes have been seen in numerous other studies by other groups as well and underscore the timely and critical need to improve DC vaccine regimens to elicit even more robust and durable immunotherapy.

In our current study we aimed to overcome the intrinsically shortened lifespan of DCs that may be limiting the efficacy and durability of DC vaccines. It is well established that DCs are short-lived species that undergo apoptosis after only 2–3 days *in vitro* and *in vivo* upon maturation [3,4]. Furthermore, cancer cells themselves may also secrete factors such as IL-10 that lower DC survival by suppressing the anti-apoptotic genes *Bcl-2*, *Bcl-*_*XL*_, and *Bfl-1*[1]. Other groups have also shown that DC longevity is an important factor in overall DC functionality [2,20,25,26]. Interestingly, Yoshikawa *et al*. demonstrated that immunization of mice with DCs infected with a Bcl-_XL_-encoding adenovirus effectively generated more potent anti-tumor immunity [26]. Even more strikingly, Park *et al.* utilized AKT-1, a known oncogenic protein, to prolong the survival of DCs [20]. In that study, AKT-1 was shown to operate via the modulation of Bcl-2 and long-lived DC vaccines were potent enough to suppress tumor growth in the therapeutic setting and significantly increased mice survival. These studies have shown that long-lived DCs may indeed represent an important avenue to pursue for future DC vaccines.

Here we sought to engineer DC vaccines that are loaded using LVs that encode both the TAA of interest and a known survival factor. We reasoned that LVs are more suitable for extending DC longevity than adenoviral vectors owing to their stable integrative nature. To this end, we engineered novel bicistronic LVs that encode the TAA, rHER-2, and one of several candidate survival factors, c-FLIP_S_, c-FLIP_L_, Bcl-_XL_, M11L, and AKT-1. As expected, both c-FLIP isoforms, Bcl-_XL_, and M11L delayed apoptosis in DC2.4 cells when cultured in low FCS media. Unexpectedly, AKT-1 did not exhibit a protective effect in our hands. A likely scenario is that the over-expressed AKT-1 was not properly activated in this context. It is well known that the activation of AKT-1 is dependent on the activation of PI3K and is also kept under control by PTEN tumor suppressor [27]. Thus, we suspect that the activation of AKT-1 was curtailed by negative feedback mechanisms in these transduced cells. Finally, in accordance with their molecular pathways [9], c-FLIP isoforms protected our transduced DC2.4s from the extrinsic cycle of apoptosis via anti-CD95 antibody activation. As well, Bcl-_XL_ and M11L protected DC2.4s from the intrinsic cycle of apoptosis as assessed via STS activation.

In this report, we also confirmed that LV transduction (using one of our novel vectors specifically) did not alter the morphology, phenotype, or function of DC2.4s and BMDCs, consistent with findings from our previous study [19]. Here we also showed that DC2.4 cells could be matured using recombinant murine IFN-γ and that once matured, both NT and transduced cells exhibited increased expression of CD80, CD86, and MHC Class II molecules [28] and increased secretion of IL-6 and TNF-α [29] consistent with known features of DC maturation. Similarly, we showed that LV-transduced BMDCs were indistinguishable from NT BMDCs in terms of DC immune surface markers in the respective immature and mature states.

## Conclusion

In our current study we generated and produced several novel candidate bicistronic LVs that may be used to load DCs and produce DC vaccines that are both TAA-specific and resistant to apoptosis. Indeed, we were successful in blocking apoptosis triggered by either the extrinsic or the intrinsic cycle in this context. Interestingly, it is currently not well known which cycle dominates in DCs and the novel vectors we have engineered in our current study can help decipher this key mechanistic insight that will lead to further improvement of future DC vaccines and (hopefully) more robust and durable objective tumor regressions.

## Methods

### Generation of LV transfer vectors encoding rHER-2 and survival factors

In order to obtain murine material for subcloning of various anti-apoptotic proteins/survival factors, we isolated C57BL/6 mouse spleen cDNAs using conventional TRIzol® RNA extraction (Invitrogen, Grand Island, NY, USA) followed by Reverse Transcriptase-Polymerase Chain Reaction (PCR) using the ThermoScript™ RT-PCR System (Invitrogen, Grand Island, NY, USA) as per the manufacturer’s instructions. To amplify survival factor cDNAs for TA Cloning (see below), PCR was performed using primers designed to amplify survival factor coding sequences (CDS) as catalogued by the National Center for Biotechnology Information (NCBI). The cDNAs for c-FLIP_S_, c-FLIP_L_, Bcl-_XL_, and AKT-1 were amplified from the isolated cDNA collection, while the cDNA for M11L was previously isolated [14] and was similarly amplified. Forward primers were strategically designed to contain the Kozak consensus sequence and either the restriction sites *EcoRV* or *StuI* flanking the 5’ end of the primer for subsequent subcloning purposes. Similarly, the restriction enzyme *NotI* was added to the 5’ end of the reverse primers to facilitate subsequent subclonings. The following primers were used to amplify the cDNAs of interest: Table 1.

**Table 1:** Primers for PCR amplification of survival gene cDNAs

**ÉcDNA**	**Primer direction**	**Primer sequence**
c-FLIP_S_	Forward	5’- CGCGATATCGCCACCATGGCCCAGAGCCCTGTGTCTG - 3’
	Reverse	5’- ATAAGAATGCGGCCGCTCATGCTGGTACTCCATACACTGGCTC - 3’
c-FLIP_L_	Forward	5’- GAAGGCCTGCCACCATGGCCCAGAGCCCTGTGTCT - 3’
	Reverse	5’- ATAAGAATGCGGCCGCTCACGTAGGAGCCAGGATGAGTTTC - 3’
Bcl-_XL_	Forward	5’- CGCGATATCGCCACCATGTCTCAGAGCAACCGGGAGC - 3’
	Reverse	5’- ATAAGAATGCGGCCGCTCACTTCCGACTGAAGAGTGAGCC - 3’
M11L	Forward	5’- AGGCCTGCCACCATGATGTCTCGTTTAAAGACGGCCG - 3’
	Reverse	5’- GCGGCCGCCTAGGTCCCTCGGTACCATTTTAGTATTCC - 3’
AKT-1	Forward	5’- GATATCGCCACCATGAACGACGTAGCCATTGTGAAGG - 3’
	Reverse	5’- GCGGCCGCTCAGGCTGTGCCACTGGCTG - 3’

PCR was performed using Platinum Taq DNA Polymerase (Invitrogen, Grand Island, NY, USA). PCR products were then purified using the QIAquick PCR Purification Kit (Qiagen, Venlo, Netherlands) and TA subcloning was accomplished using pGEM®-T Easy Vector as per the manufacturer’s instructions (Promega, Fitchburg, Wiscosin, USA).

The cDNAs for c-FLIP_S_, c-FLIP_L_, Bcl-_XL_, M11L, and AKT-1 were then directionally subcloned into the pDIG/EMCV IRES plasmid shuttle vector using the restriction enzyme sites *EcoRV/StuI* and *NotI* and resulting nucleotide sequences were confirmed by bidirectional sequencing. The fragments of DNA corresponding to IRES- [c-FLIP_S_/c-FLIP_L_/Bcl-_XL_/M11L/AKT-1] were then amplified by PCR from the respective subcloned pDIG/EMCV IRES vectors using primers designed to be flanked by either *SalI* or *PspXI* restriction enzyme sites for subsequent non-directional molecular subcloning steps. The primers used for PCR amplification were as follows: Table 2.

**Table 2:** Primers for PCR amplification of IRES-survival gene cDNAs

** Sequence**	**Primer direction**	**Primer sequence**
IRES-c-FLIP_S_	Forward	5’- ATAACTCGAGTGCCCCTCTCCCTCCCCCC - 3’
	Reverse	5’- ATAACTCGAGTGCGGCCGCTCATGCTGGTA - 3’
IRES-c-FLIP_L_	Forward	5’- ATAACTCGAGTGCCCCTCTCCCTCCCCCC - 3’
	Reverse	5’- ATAACTCGAGTGCGGCCGCTCACGTAGGAG - 3’
IRES-Bcl-_XL_	Forward	5’- ATAACTCGAGTGCCCCTCTCCCTCCCCCC - 3’
	Reverse	5’- ATAACTCGAGTGCGGCCGCTCACTTCCGA - 3’
IRES-M11L	Forward	5’- GTCGACGCCCCTCTCCCTCCCCCC - 3’
	Reverse	5’- GCGGCCGCCTAGGTCCCTCGGTACCATTTTAGTATTCC - 3’
IRES-AKT-1	Forward	5’- GTCGACGCCCCTCTCCCTCCCCCC - 3’
	Reverse	5’- GTCGACGCGGCCGCTCAGGCTGT - 3’

As described above, PCR products were then purified and TA-subcloned into the pGEM®-T Easy Vector as per the manufacturer’s instructions. The IRES- [c-FLIP_S_/c-FLIP_L_/Bcl-_XL_/M11L/AKT-1] nucleotide fragments were then subcloned into the previously engineered lentivirus SIN transfer vector pCCL-rHER-2/neu using the restriction enzyme sites *SalI* or the *SalI*-compatible restriction enzyme site, *PspXI*[19]. Importantly, pCCL-rHER-2/neu vector plasmid was digested using the *SalI* restriction enzyme and dephosphorylated using rAPid Alkaline Phosphatase (Roche, Penzberg, German) prior to T4 ligase treatment as per the manufacturer’s instructions to prevent vector self-ligation. Plasmids were sent to The Centre for Applied Genomics - The Hospital for Sick Children for bi-directional DNA sequencing for validation of nucleotide sequence conformity and absence of non-sense mutations. No anomalies were found. The aforementioned engineered transfer vector plasmids were denoted as: pCCL-rHER-2/neu, pCCL-rHER-2/neu-c-FLIP_S_, pCCL-rHER-2/neu-c-FLIP_L_, pCCL-rHER-2/neu-Bcl-_XL_, pCCL-rHER-2/neu-M11L, and pCCL-rHER-2/neu-AKT-1.

### Cell lines

HEK 293T cells (ATCC, Manassas, Virginia, USA) were cultured in Dulbecco’s Modified Eagle Medium (DMEM) (Sigma Aldrich, St. Louis, MO, USA) supplemented with 10% heat-inactivated FBS (PAA Laboratories Inc., Piscataway, NJ, USA), 100 units/mL penicillin/100 μg/mL streptomycin/ 29.2 mg/mL L-glutamine (Invitrogen Gibco, Carlsbad, CA, USA) and incubated at 37°C in 5% CO_2_- containing atmosphere. DC2.4 cells [21] were cultured in Roswell Park Memorial Institute (RPMI-1640) media (Sigma Aldrich, St. Louis, MO, USA) supplemented with 10% heat-inactivated FCS Gold (PAA Laboratories Inc., Piscataway, NJ, USA), 100 units/mL penicillin, 100 μg/mL streptomycin, 29.2 mg/mL L-glutamine (Invitrogen Gibco, Carlsbad, CA, USA), and 0.1 mM 2-mercaptoethanol and incubated at 37°C in a 5% CO_2_ – containing atmosphere.

### Production of LVs and transduction of cell lines

Lentiviral vectors pseudotyped with vesicular stomatitis virus-glycoprotein (VSV-g) were generated from the transient co-transfection of the newly-engineered SIN transfer vector, second-generation LV packaging construct pCMVΔ8.91 [30], envelope plasmid pMD.G [31], and pAdVAntage™ vector [32] (Promega, Madison, WI, USA) into HEK 293T cells (ATCC, Manassas, VA, USA) using polyethyleneimine (PEI) as previously described [33]. Virus supernatants were harvested 48 hours and 72 hours after transfection, filter-sterilized using 0.22-μm filters (Nalgene, Rochester, NY, USA), and concentrated at 50,000 X g for 2 hours using an Optima L-100 XP Ultracentrifuge (Beckman Coulter, Brea, CA, USA). LV pellets were re-suspended in serum-free X-VIVO™ 20 medium (BioWhittaker, Basel, Switzerland). Functional titers were determined by serially-diluted LV transductions of HEK 293T cells, followed by subsequent flow cytometry analyses for HER-2 expression. Typical functional titers of ultracentrifuge-concentrated LVs used in this study were in the range of 5 x 10^7^ to 1 x 10^8^ IU/mL. The following antibodies were used for flow cytometry: anti-c-ErbB2/c-neu (Ab-4) Mouse mAb (Calbiochem, Darmstadt, Germany) and goat anti-mouse IgG-FITC (Santa Cruz Biotechnology, Dallas, TX, USA).

Cells were plated onto 6-well tissue culture-treated plates 1 day prior to transduction. On the day of transduction, LV volumes corresponding to the desired MOI were diluted in 1 mL of complete media containing 8 μg/mL of protamine sulfate (Sigma, St. Louis, MO, USA), added to the cells, and incubated for 24 hours at 37°C with 5% CO_2_. Following the incubation period, the protamine sulfate containing-media was replaced with fresh complete media. Forty-eight hours after media change, the cells were either collected for analysis of transduction efficiencies and/or passaged for subsequent experimentation.

### Immunoblot assays to assess expression of survival factors

Western blots were performed to confirm the expression of the downstream survival factors. Transduced HEK 293T/DC2.4 cells were harvested and lysed using RIPA lysis buffer (Thermo Scientific Pierce, Waltham, MA, USA); subsequent protein concentrations were quantified using BioRad DC Protein Assay (BioRad, Hercules, CA, USA). Next, 50 μg of protein was loaded onto each lane and 10% Sodium Dodecyl Sulfate (SDS) – PolyAcrylamide Gel Elecrophoresis (PAGE) was performed at 100 Volts (V) for 100 minutes. Proteins were then transferred onto Polyvinylidene fluoride (PVDF) membranes (Thermo Scientific Pierce, Waltham, MA, USA) using the Biorad Semi-Dry Electrophoretic Transfer Cell at 30V for 30 minutes (Biorad, Hercules, CA, USA). Following transfer, membranes were washed in 0.1% Tween-20-PBS (TPBS) and blocked overnight at 4°C with 5% non-fat dried milk (NFDM) freshly prepared in TPBS. Membranes were probed with primary antibody in TPBS/2.5% NFDM overnight at 4°C. The following primary antibodies were used at the specified dilutions: anti-β-actin (Millipore, Billerica, MA, USA, 1:1000), anti-c-FLIP G-11 (Santa Cruz Biotechnology, Dallas, TX, USA, 1:500), anti-Bcl-_XL_ Clone 44 (BD Biosciences, Franklin Lakes, NJ, USA, 1:500), rabbit anti-M11L serum (previously generated [34], 1:500), and anti-AKT1 G-5 (Santa Cruz Biotechnology, Dallas, TX, USA, 1:1000). Following primary antibody incubation, membranes were washed and probed with either anti-rabbit IgG-HRP (Santa Cruz Biotechnology, Dallas, TX, USA, 1:10000) or anti-mouse IgG-HRP (GE Healthcare, Waukesha, WI, USA, 1:10000) secondary antibody diluted in TPBS/2.5% NFDM. Membranes were then developed using ECL reagents (Thermo Scientific Pierce, Waltham, MA, USA) and exposed onto autoradiograph film.

### WPRE Real-Time PCR assay

Genomic DNA was extracted from harvested DC2.4 cell lines using the Gentra Puregene Blood Kit (Qiagen, Venlo, Netherlands) according to the manufacturer’s instructions. Following extraction, DNA yields and concentrations were determined using a Thermo Scientific NanoDrop for subsequent PCR reactions. Real-time PCR was then performed as previously described [22] using the Corbet Research Rotor Gene RG3000 thermo-cycler based on the Taqman system. Each PCR reaction was performed in conjunction with a no-template reaction negative control. Each DNA sample was tested in triplicate and mean values were reported. Standard curves were generated based on serial dilutions (five dilution points) of genomic DNA from a 293T cell line containing one provirus (one copy LV transduction was previously established in our laboratory and its one copy status was confirmed by Southern blot analysis [22]) added into isolated genomic DNA from a confirmed NT 293T cell line. Assay sensitivity was determined to be as low as 324 vector-positive cells per 1 million 293T cells (data not shown).

### Assessment of cell death following FCS withdrawal, anti-CD95 addition, and STS treatment

In the DC2.4 FCS reduction experiments, DC2.4 cells were transduced at a functional MOI of 20, yielding transduction efficiencies ranging from 8.8-23.6% (data not shown) and sorted on a MoFlo Beckman Coulter Cell Sorter based on positive rHER-2/neu expression. Staining here for rHER-2/neu was performed as above. Sorted cell populations were then expanded and deprived of growth factor in DC2.4 apoptosis-inducing media (RPMI-1640 supplemented with 2% FCS, 100 IU/mL penicillin, 100 μg/mL streptomycin, 1% L-glutamine, 0.1% 2-mercaptoethanol). Cells were then plated onto 6-well tissue culture-treated plates at a density of 5 x 10^4^ DC2.4 cells per well in 1.5 mL volume of apoptosis-inducing media for 48 hours and subsequently analyzed by flow cytometry following Annexin V/PI staining (BD Biosciences, Franklin Lakes, NJ, USA). Each treatment group was plated in triplicate for statistical evaluation and compared to the same DC2.4 cell lines grown in complete media (RPMI-1640 supplemented with 10% FCS, 100 IU/mL penicillin, 100 μg/mL streptomycin, 1% L-glutamine, 0.1% 2-mercaptoethanol). Relative cell death was defined by Annexin V+/PI+ double-positive populations representing apoptotic cell death and normalized to 10% treatment groups i.e. Relative Cell Death = % of total cell population expressing Annexin V and PI in treatment group / % of total cell population expressing Annexin V and PI in 10% treatment group).

To activate the extrinsic cycle of apoptosis, previously transduced and sorted DC2.4 cells were similarly plated onto 6-well tissue culture-treated plates at a density of 5 x 10^4^ DC2.4 cells per well in 1.5 mL volume of complete media (RPMI-1640 supplemented with 10% FCS, 100 IU/mL penicillin, 100 μg/mL streptomycin, 1% L-glutamine, 0.1% 2-mercaptoethanol) with either the anti-CD95 Clone Jo2 antibody (BD Biosciences, Franklin Lakes, NJ, USA) or the isotype control Clone Ha4/8 (BD Biosciences, Franklin Lakes, NJ, USA) added at a dilution of 1 μg/ml as per the manufacturer’s instructions. Likewise, to activate the intrinsic cycle of apoptosis, cells were similarly plated in complete media and treated with or without STS (Invitrogen, Grand Island, NY, USA) as per the manufacturer’s instructions at 1 μM. Each treatment group was plated in triplicate for statistical evaluation. Apoptosis and cell death was subsequently analyzed by flow cytometry following Annexin V/PI staining. Relative cell death as defined by Annexin V+/PI+ populations were normalized to isotype control and no STS treatment groups, respectively (i.e. Relative Cell Death = % of total cell population expressing Annexin V and PI in treatment group / % of total cell population expressing Annexin V and PI in isotype control or no STS treatment group).

### DC2.4 and BMDC maturation immuno-phenotypic analysis

DC2.4 cell lines were cultured as described above and matured using recombinant murine IFN-γ (R&D Systems, Minneapolis, MN, USA) at a concentration of 100 ng/mL for 24 hours and subsequently harvested for flow cytometry analyses. BMDCs were cultured, differentiated, transduced, and matured as previously described [19]. On day 7 of culture, BMDCs were harvested for flow cytometry analyses. Fc receptors were blocked using purified anti-mouse CD16/CD32 Clone 2.4G2 (BD Biosciences). The following antibodies were used for flow cytometry: anti-mouse CD11c Clone N418 (eBioscience, San Diego, CA, USA), Armenian Hamster IgG isotype Clone eBio299Arm (eBioscience), anti-mouse CD86 Clone GL1 (BD Biosciences), IgG2a_κ_ isotype Clone R35-95 (BD Biosciences), anti-mouse CD80 Clone 16-10A1 (BD Biosciences), Hamster IgG2_κ_ isotype Clone B81-3 (BD Biosciences), anti-mouse I-A[b] Clone AF6-120.1 (BD Biosciences), Mouse IgG2a_κ_ isotype Clone G155-178 (BD Biosciences). rHER-2 staining was performed as described above for DC2.4 cells.

### DC2.4 cytokine secretion analysis

Twenty-four hours after maturation, culture supernatants were collected for cytokine secretion quantification. Murine IL-6 and murine TNF-α were detected using the BD OptEIA ELISA kits (BD Biosciences) as per the manufacturer’s instructions.

### Statistical analyses

Experiments were performed at least twice independently unless otherwise stated and representative data are shown with error bars representing standard deviations. For statistical comparison of treatment groups, two-tailed unpaired Student’s t-tests were performed. *p* values < 0.05 were designated as statistically significant (*).

## Abbreviations

BMDC: Bone marrow-derived dendritic cells; DC: Dendritic Cell; c-FLIP: Cellular FLICE Inhibitory Protein; EMCV: Encephalomyocarditis Virus; FCS: Fetal Calf Serum; FADD: Fas-Associated Death Domain; IRES: Internal Ribosomal Entry Site; LV: Lentivector; MOI: Multiplicity of Infection; NT: Non-Transduced; rHER-2: rat Human Epidermal growth factor Receptor 2; STS: Staurosporine; TAA: Tumor-Associated Antigen; TRAIL: TNF Related Apoptosis-Inducing Ligand; WPRE: Woodchuck Hepatitis Virus Post-transcriptional Regulatory Element.

## Competing interests

The authors declare no potential conflicts of interest.

## Authors’ contributions

JCMW, TCF, and BCYA performed experiments. JCMW, TCF, DHF, GAD, and JAM participated in the design of the study. JCMW and JAM wrote the manuscript. All authors read, edited, and approved the final manuscript.
